# Supplementary material for: Antidiabetic Effects of Bisamide Derivative of Dicarboxylic Acid in Metabolic Disorders
Source: Int J Mol Sci. 2020 Feb 3;21(3):991. doi: 10.3390/ijms21030991 (PMC7037053; doi:10.3390/ijms21030991)
Supplement: Supplementary file 1 [file ijms-21-00991-s001.pdf]

Table S1 Supplement: The body mass index (BMI) of male C57BL/6 mice on 70th day of the experiment (M±m, n=10)

Index	Intact control (group 1)	Metabolic disorders (group 2)	Mice with metabolic disorders treated by BDDA (group 3)
BMI	0.233±0.003	0.306±0.004*	0.241±0.004●

* - $p<0.05$ in comparison with intact control, ● - $p<0.05$ in comparison with metabolic disorders
